# Preliminary Research on Response of GCr15 Bearing Steel under Cyclic Compression

**DOI:** 10.3390/ma13163443

**Published:** 2020-08-05

**Authors:** Xiaomeng Zheng, Yongzhen Zhang, Sanming Du

**Affiliations:** National United Engineering Laboratory for Advanced Bearing Tribology, Henan University of Science and Technology, Luoyang 471003, China; dbf@haust.edu.cn (X.Z.); dsming@haust.edu.cn (S.D.)

**Keywords:** bearing steels, rolling contact fatigue, microstructure, cyclic-compression, non-metallic inclusion

## Abstract

During the bearing service, a series of microstructural evolutions will arise inside the material, such as the appearance of feature microstructures. The essential reason for the microstructural evolution is the cumulative effect of cyclic stress. The Hertz Contact formula is usually adopted to calculate the internal stress, and there is a correlation between the shape and distribution of the feature microstructure and the stress distribution. But it is insufficient to explain the relationship between the morphology of feature microstructures and the rolling direction, such as specific angles in butterfly and white etching bands. The rolling phenomenon will cause the asymmetry of stress distribution in the material, which is the source of the rolling friction coefficient. Moreover, slipping or microslip will produce additional stress components, which also cause the asymmetry of the stress field. However, there is no experimental or theoretical explanation for the relationship between the asymmetry of the stress field and the feature microstructure. According to the current theory, the appearance of feature microstructures is caused by stress with or without rolling. Therefore, it is of great significance to study the formation mechanism: whether feature microstructures will appear in the uniaxial cyclic compression stress field without rolling. In this paper, uniaxial cyclic compressive stress was loaded into a plate-ball system and a cylinder system. The characteristics of microstructural change of bearing steel (GCr15) were studied. It was found that the hardness of the material increased after the cyclic compressive load, and the inclusions interacted with the matrix material. In the local microregion a white etching area was found, although the scale is very small. No large-scale feature microstructures appeared. Other phenomena in the experiment are also described and analyzed. For example, the production of oil film in the contact area and the changing law of alternating load.

## 1. Introduction

For decades, the evolution of the internal microstructure of bearing materials in service has been one of the hot spots in bearing research. Especially, the mechanism of feature microstructures has attracted much attention in recent years. Up to now, four kinds of feature microstructures, butterfly, white etching cracks (WECs), dark etching region (DER) and white etching bands (WEBs) have been found. The first two are related to cracks while the others are not [1]. Butterfly and WECs are white etching area (WEA) associated microstructures produced by the interaction of materials on both sides of the crack or inclusion-matrix interface under cyclic stress. WEA is the result of phase transition caused by contact or microfriction [2,3,4,5]. DER and WEBs are ferrite-carbide intergrowth microstructures produced by stress-induced carbon migration assisted with dislocation movement [6,7,8,9,10]. No matter which feature microstructure it is, it is the result of stress-induced phase transition, which has been recognised and accepted by many scholars [4,5,11,12]. The reason is that the service condition of the bearing is generally below 70 °C, which belongs to the normal temperature range for steel materials. Although temperature [13,14] and friction heat [15,16,17,18] can promote the appearance of feature microstructures, they are not the essential reason [5].

It is found that the morphology of the butterfly is directly related to the rolling direction of the rolling body, and the 45° crack always faces the rolling direction. The four wing butterfly can be obtained by rotating the bearings in reverse direction after a period of normal rotation [19]. WEBs also present a certain angle with the rolling direction. According to the research results of Fu [10], the generation of DER is the cumulative result of orthogonal shear stress. Based on current theories and mathematical models, it can be speculated that DER will arise as long as there is an alternating orthogonal shear stress in the material.

The radial load on the bearing causes the nonconforming elastic contact between the inner ring and the rolling element. Under static load, the internal stress of the bearing material can be calculated by Hertz Contact theory. The stress field in the material is symmetrical with the contact point. For a certain point in the inner ring, the root cause of its stress history is the motion of the Hertz Contact stress field along the material’s surface. Regardless of the rolling direction, the stress history caused by the symmetrical stress field is the same. When tangential traction is introduced into the contact surface, the distribution of the internal stress field will be changed [20], resulting in obvious asymmetry. The stress history presents correlation with the traction direction [21]. The tangential friction of the surface is produced by roll-slip ratio, microsliding or static friction. In addition, in the rolling process, the material in front of the contact point has the tendency of compression, and the material behind the contact point has the tendency of rebound. The difference of the deformation state will also cause the asymmetry of stress distribution, and it then causes the correlation between stress history and rolling direction.

The purpose of this paper is to study the change of microstructure in materials under uniaxial cyclic compressive load. The uniaxial cyclic compressive load can also produce alternating stress fields in the material, and the effect of stress asymmetry caused by tangential friction and rolling on the surface is eliminated. It is of great significance to study the formation mechanism of feature microstructures.

In order to simulate the service characteristics of the bearings as much as possible, the plate-ball system is used to undergo the cyclic compression load. In this system, a typical Hertz Contact will be produced, which is the same to the contact form of the bearings internal components. It is well known that the Hertz Contact causes complex nonuniform stress fields in materials. It is interesting whether the appearance of feature microstructures is related to the characteristics of stress distribution. Therefore, a cylindrical system, in which the uniform stress field can be obtained, is added to compare the material responses under uniform and nonuniform stress fields.

## 2. Experimental Methods

### 2.1. Materials

GCr15 (ASME 52100) bearing steel was prepared as the target material. The chemical composition is listed in Table 1 and was detected by CITIC Heavy Industry (Luoyang, China) through an optical emission spectrometer. The hardness of the material was enhanced to HRC63 by heat treatment. It was austenitised at 845 °C for 10 min, oil-quenched, then, tempered at 150 °C for 3 h, obtaining a tempered martensite microstructure.

### 2.2. Experiment

**Figure 1:**
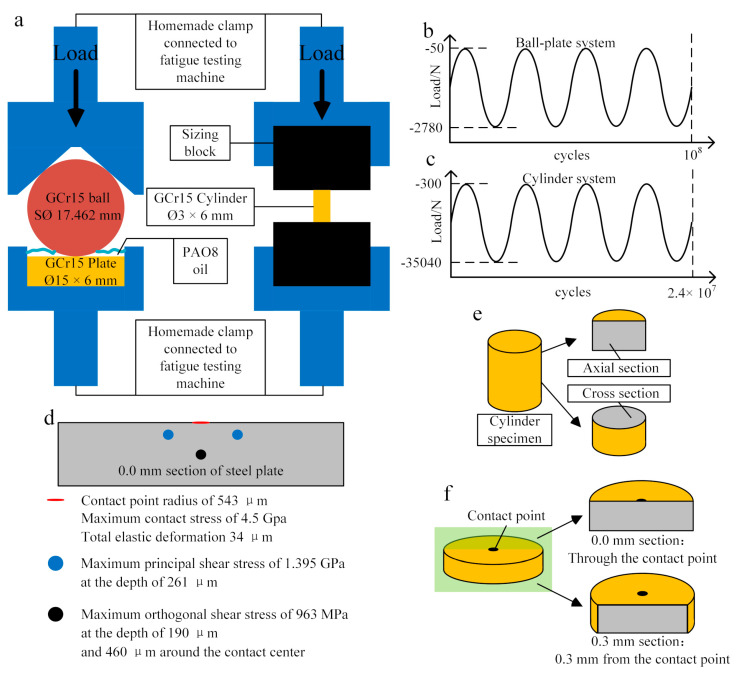
Experimental details (**a**) schematic of plate-ball and cylinder systems; (**b**) the cyclic compressive load exerted on the plate-ball system; (**c**) the cyclic compressive load exerted on the cylinder system; (**d**) internal stress details of steel plate in plate-ball system; (**e**) observation section of cylinder specimen (related to Figure 7); (**f**) observation section of plate specimen (related to Figures 8, 10–13, 16 and 17).

Two experimental forms were used in the study, as shown in Figure 1. The steel material was cut into plate (Ø15 × 6) and cylinder (Ø3 × 6), then mechanically polished into a mirror surface on one or two end planes to bear load. A GCr15 steel ball (G10 grade, SØ 17.462) was selected as mating material for the plate specimen. An electromagnetic resonance, high-frequency fatigue testing machine (GPS-200) was used as experimental equipment to induce the cyclic compression load. The ball and plate specimen were assembled into a homemade clamp to limit the lateral movement. In order to prevent impact, the minimum force of alternating load was set as the non-zero value in the experiment.

In order to produce a maximum contact stress (*P_0_*) of 4.5 GPa in the material, 50–2780 N cyclic compression load was employed in the plate-ball system. In this case, normal pressure introduced a contact radius of 543 μm, a maximum principal shear stress of 1.395 GPa at the depth of 261 μm, a maximum orthogonal shear stress of 963 MPa at the depth of 190 μm and 460 μm around the contact center [22,23,24,25,26,27]. Since no orthogonal shear stress exists in the cylinder specimen, the normal load of 300–35,040 N was employed in the cylinder system, causing a normal stress of 2.79 GPa. The same maximum principal shear stress of 1.395 GPa was obtained in the two systems.

The whole experiment was carried out consecutively at an ambient temperature of 19 °C. The total stress cycle was 10^8^ and 2.4 × 10^7^ for the plate-ball system and cylinder system, respectively. In addition, the contact point in the plate-ball system was immersed in PAO8 oil. This was to make the experiment more in-line with the working condition of the bearing.

### 2.3. Measurement and Observation

The post-test plate specimen was cut along the axial section, and the post-test cylinder specimen was cut along the axial section and cross-section (Figure 1). For inclusions observation, the specimens were ground with 1200# SiC sand paper and polished with 3.5 μm, 2.5 μm, 1μm diamond spray and 0.04 μm OP-S sequentially. To show the microstructure of the material, 4% nitric alcohol solution was used. The microscopic observation was achieved using a field emission scanning electron microscope (FESEM JSM-7800F, JEOL, Tokyo, Japan) equipped with energy dispersive spectra (EDS) detection and a tungsten filament scanning electron microscope (SEM JSM-IT100, JEOL, Tokyo, Japan). A nonconforming stress field was introduced by Hertz Contact in the plate-ball system. Before the microscopic observation, an optical microscope was employed to locate the position of nonmetallic inclusions using the image mosaic method, after which specimens were etched. A Micro Vickers hardness tester (HV-1000, Laizhou Huayin Testing Instrument Co., Ltd., Laizhou, China) was used to reveal the hardness change before and after test. Indentations caused by cyclic compression load on both plate and ball were scanned using a 3D Surface Profilometer (Nanofocus AG, Oberhausen, Germany) to investigate the shape, depth and surface roughness.

## 3. Results and Discussions

### 3.1. Alternating Load

The cyclic compression load was automatically applied by the equipment (GPS-200, Sinotest, Changchun, China). The frequency of load depends on the stiffness of the equipment and the specimens. In this experiment, the specimens are not rigidly fixed, the frequency will change during the experiment, as well as the load.

**Figure 2:**
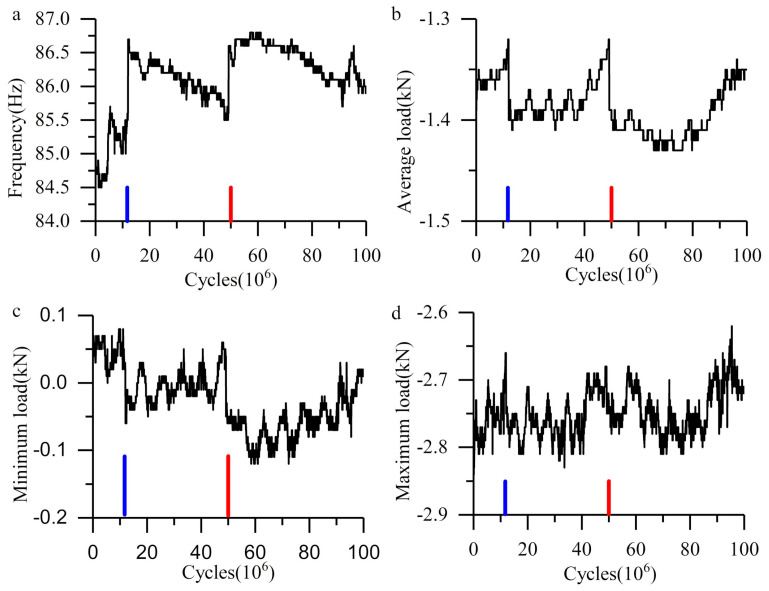
Load and frequency curve in the plate-ball system. (**a**) Frequency curve; (**b**) average load curve; (**c**) minimum load curve; (**d**) maximum load curve.

Figure 2 shows the load and frequency variation during the experiment of plate-ball system. As mentioned above, the load range is 50–2780 N, however, the actual load has been lower than 50 N, and even changed to tensile stress. As the plate and ball is separated, the tensile stress is the display problem of the sensor, not the real tensile stress. This separation results in the stiffness decreasing in the whole system. The Hertz Contact of the plate-ball will also contribute to this stiffness change. At the initial stage of the experiment, the contact radius of the plate-ball is small and the ratio of elastic deformation is greater than that of plastic deformation. With the progress of the experiment, the plastic deformation inside the material accumulates, resulting in expansion of the actual contact area and the increase of stiffness. This explains why the load and frequency increase at 10 × 10^6^ cycles (blue lines in Figure 2). The change of frequency indicates the accumulation of plastic deformation in the material. As for the sudden change at another time (red lines in Figure 2), it is because the ball has the freedom of rotation during the experiment. The rotation of the ball makes a new part of its surface contact with the steel plate, so the plastic deformation accumulates again. The contact mark on the ball surface is less than 30 mm long; however, the whole experiment lasted for 326 h. And the rotation only occurs when the load is above zero. Therefore, the friction caused by rotation can be ignored. The maximum load, the average load and the minimum load show the same change rule, which means that the vibration of the cyclic compression stress is the translation of the whole load spectrum.

**Figure 3:**
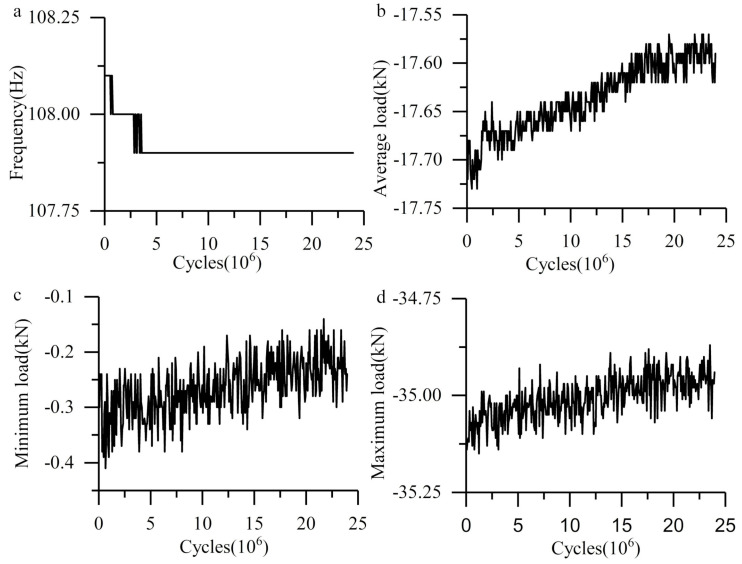
Load and frequency curve in cylinder system. (**a**) Frequency curve; (**b**) average load curve; (**c**) minimum load curve; (**d**) maximum load curve.

The load and frequency curve in the cylinder system are shown in Figure 3. Compared with the plate-ball system, the variation is more stable, which benefits from the stiffness of the whole system. The whole load spectrum shifts, and the frequency remains stable until the end of the experiment after early fluctuations. Nevertheless, the degree of change is very small, which means the plastic deformation was very weak.

### 3.2. Contact Point in Plate-Ball System

According to Hertz Contact theory, the radius of the contact area and the whole elastic deformation of the plate-balls system can be calculated as 543 μm and 33.7 μm. Theoretically, the maximum internal stress produced by the normal pressure in the experiment does not exceed the yield limit of the material. In fact, due to the nonuniformity and surface roughness of materials, plastic deformation occurs in the interior of the material, but it is very weak in each load cycle. Similar to the service process of bearing, after sufficient cycles, obvious plastic deformation traces appear on the surface of the material.

**Figure 4:**
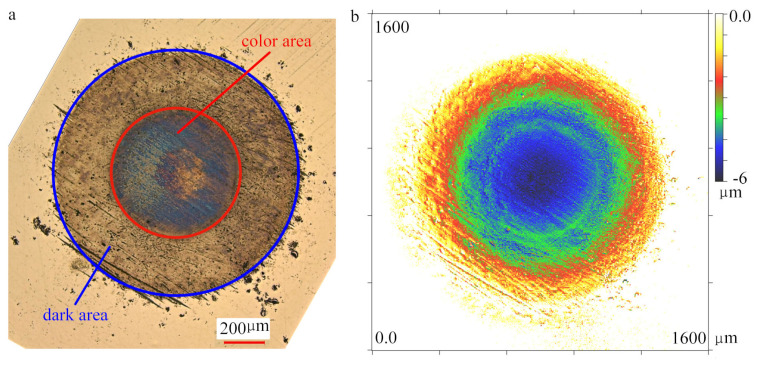
Photomicrograph and 3D profile of contact point on the plate. (**a**) Micrograph of optical microscope; (**b**) surface topography.

**Figure 5:**
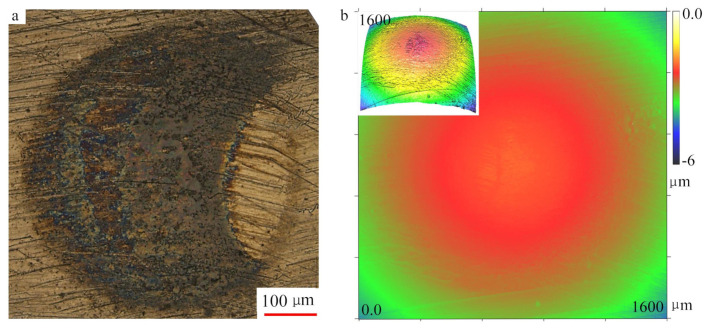
Photomicrograph and 3D profile of the contact point on the ball. (**a**) Micrograph of optical microscope; (**b**) surface topography.

Figure 4 reveals surface morphology and profile of the contact point on the plate. Figure 4a was reconstructed through extended depth of focus, three regions can be seen inside the visual field. The outermost area (outside the blue circle) is the noncontact region, the dark area (region between blue and red circle) is the contact region without colour, the center area (inside the red circle) is the contact region with colour. The whole contact area was measured to be a circle of radius 601 μm, slightly greater than the theoretical value. The depth of the indentation was about 6 μm, see Figure 4b. The colour region inside the contact area in Figure 4a was presumably lubricating oil film. The surface roughness of the colour area (Ra 0.1–0.2) is less than that of the dark area (Ra 0.22–0.32). The contact point on the ball shows a similar morphology (Figure 5). Due to the rotation of the ball during the experiment, the area of contact point on the ball surface is not a complete circle, but the outline still can be seen, which corresponds to the colour area in Figure 4a, because they have the same radius. Additionally, no obvious shape change was observed on the surface of the ball. It shows that most of the plastic deformation occurs in the plate, see Figure 4b and Figure 5b.

**Figure 6:**
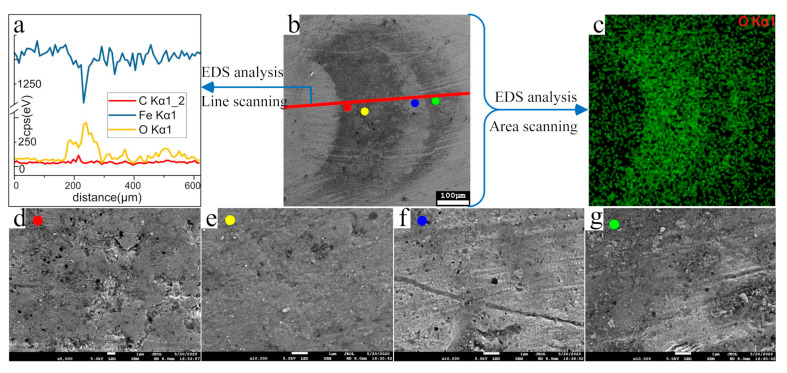
SEM image and EDS detection element distribution of contact point on the ball. (**a**) Element distribution along the red line shown in b; (**b**) SEM image; (**c**) oxygen distribution in the area of **b**; (**d**) SEM photomicrograph of the position of red spot in b; (**e**) SEM photomicrograph of the position of yellow spot in b; (**f**) SEM photomicrograph of the position of bule spot in b; (**g**) SEM photomicrograph of the position of green spot in b.

The results of SEM and EDS (Figure 6) show that the contact area on the ball contains more oxygen (Figure 6a–c) and presents a smoother surface (Figure 6d–g). During the experiment, the contact point of the plate-ball is immersed in the lubricating oil, so the oxygen in the air cannot make contact with the metal, and the main chemical component of the oil is carbon hydrogen and oxygen. The ball was wiped with alcohol absorbent cotton and soaked in alcohol for ultrasonic cleaning before SEM observation. Therefore, it can be judged that under cyclic compression, the metal and oil react to form an oil film. Only immersing the bearing steel in lubricating oil will not produce oil film. Under the action of stress can the oil film appear, such as the cyclic compressive stress mentioned in this paper. The stress promotes the reaction between lubricating oil and metal. It is worth noting that not all contact areas have oil film, which means the oil film generation needs to meet certain conditions, for example a suitable compressive stress range.

### 3.3. Hardness

In the process of bearing service, not only the structural evolution arises, but also the hardness of the material changes gradually. The general rule of hardness evolution is to increase first and then decrease. After the appearance of feature microstructure, the hardness decrease is obvious, which is lower than the original hardness of the material [6,10,28,29,30]. The early strengthening stage is due to the accumulation of local plastic deformation, which is essentially known as cold work hardening, the decrease of hardness is caused by the transformation of martensite to ferrite. In addition, the degree of hardness change is directly related to the internal stress of the material.

**Figure 7:**
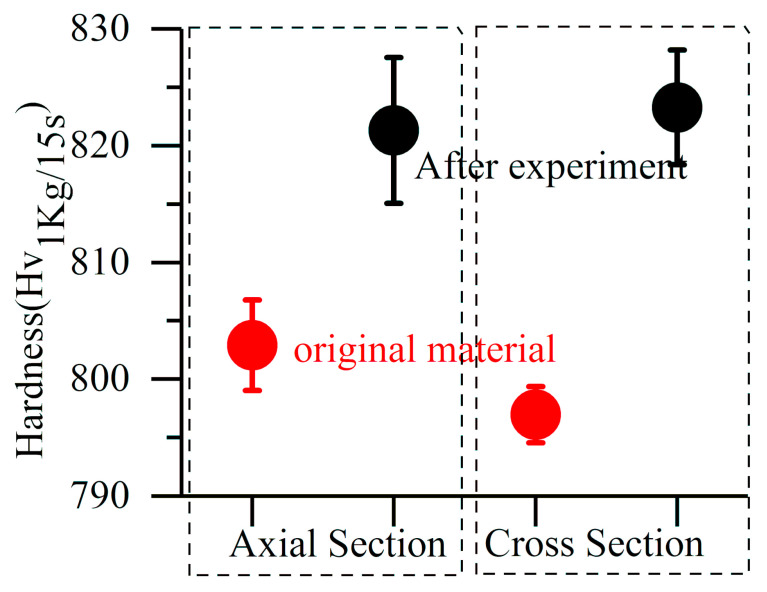
Hardness change of cylinder sample before and after experiment.

In this experiment, the hardness of the cylinder specimen is improved after cyclic stress. However compared with the material after rolling contact [29], the improvement is very slight, as Figure 7 shows. In theory, most bearing materials will not yield under service conditions. After millions of cyclic stresses, the tiny microstructural changes in the material gradually accumulate to a certain order of magnitude. This will cause visible or detectable alteration in the microstructure and properties. The tiny microstructural changes refer to the local plastic deformation at micron/nano level or even the generation and movement of dislocations. The slight hardening phenomenon found in the experiment is just the cumulative result of cyclic compression stress.

**Figure 8:**
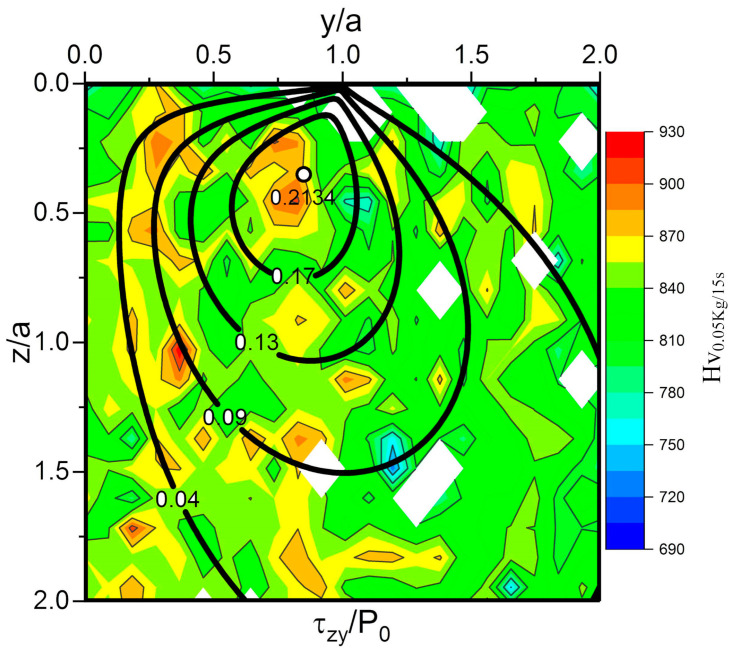
Hardness change of plate specimen at 0.0 mm section after experiment, detection interval was 50 μm. Drawing of stress contours is based on Fu’s thesis [10]. For a point inside the material, y/a is the ratio of the horizontal distance (from the point to the contact center) to the contact radius, and z/a is the ratio of the depth to the contact radius.

In the plate specimen, significant change of hardness was observed, as shown in Figure 8. This diagram combines the orthogonal shear stress (τ_zy_) distribution and hardness test results of 0.0 mm section (axial section through contact point), and the relationship between them can be shown intuitively. It can be seen that there is a corresponding relationship between the increase of internal hardness and the distribution of orthogonal shear stress, although it is not very strong. The hardening of the material is due to plastic deformation. In general, the yield strength of the material relates to the principal shear stress (3rd strength theory) or the Mises stress (4th strength theory). However, according to the results of this paper, the plastic deformation is the result of orthogonal shear stress. Orthogonal shear stress plays an important role in the service process of bearing. For example, rolling contact fatigue theory, formation of DER (described in detail in Section 3.4.1) and formation of butterfly. The rolling contact fatigue life calculation method proposed by Lundberg and Palmgren considered the effect of orthogonal shear stress on materials [31,32]. Therefore, the findings of this study are also reasonable. Lastly, the degree of hardness increase is greater than that under rolling contact condition [29], that is, under the similar contact stress and similar cycles, the increase rate of hardness is larger in this experiment.

### 3.4. Microstructure

#### 3.4.1. No DER Formation

**Figure 9:**
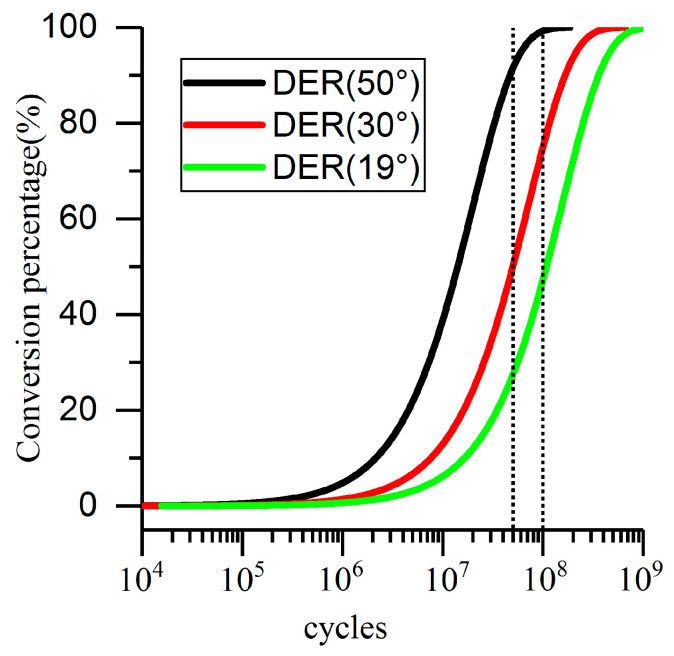
Effects of cyclic stress and temperature on the dark etching region (DER) formation under the experimental conditions (4.5 GPa).

The root cause of DER is the cumulative effect of cyclic stress. Thus, according to the existing theory, under the cyclic compression, the DER still appears inside the material of the plate. According to Fu’s theory [6,7,8,9,10,33,34], the essential reason for the appearance of DER is the movement of dislocations caused by orthogonal shear stress. The influential factors are stress value and temperature. The development prediction curves of DER can be obtained by substituting the experimental parameters into the mathematical model. Figure 9 shows the formation of DER at different temperatures under 4.5 GPa. It can be seen that the higher the temperature is, the faster the development of DER. When DER reaches 25%, it can be easily observed by optical microscope [6].

During the experiment, the temperature of the ball surface was measured directly by thermocouple—the measured value was about 30 °C. Although the actual temperature of the contact point cannot be measured, the temperature range of it must be above the measured value. Conservatively speaking, the temperature of the plate specimen must be above 30 °C during the experiment. In summary, according to the existing theory and model, DER will appear in the plate specimen, and its development rate is at least 75%, see the red curve in Figure 9. However, DER was not found in the plate specimen. The microstructure of plate specimen was observed at two axial sections, one through the contact center (0.0 mm section), the other one at 0.3 mm from the contact center (0.3 mm section).

The most obvious difference between the experimental condition and the service condition of the bearing is that there is only cyclic stress in this experiment and no rolling phenomenon. What the rolling causes is microsliding friction and different stress history. In DER-related research, full fluid film lubrication will be ensured. No matter what the degree of microsliding is, the friction coefficient caused by it will not exceed that of rolling bearing itself (10^−2^ order of magnitude). The friction torque caused by microsliding is just a part of the friction torque of the whole bearing. Therefore, the influence of tangential friction on the internal stress field is very limited. Under the condition of cyclic compression, the stress value of a point inside the material increases from zero and then decreases to zero. The change of material stress can be understood as the change of stress field from zero to the maximum. Under rolling conditions, the stress field produced by Hertz Contact does not change with time, but its position moves with rolling. Therefore, the stress evolution of a certain point in the material under rolling conditions is due to the movement of the whole stress field.

The most typical example is the evolution of orthogonal shear stress. In the rolling conditions, orthogonal shear stress history is from zero to the maximum, and then to zero, and turns into the reverse maximum at last. However, under cyclic compression, the stress history is from zero to the maximum, and then becomes zero again without reverse. This means that the material undergoes two orthogonal shear stresses of the same magnitude and opposite direction in one cycle under rolling conditions, twice as much as that of the cyclic compression condition. Therefore, for the DER model, the cycles of cyclic compression stresses in this experiment should be half of the original, which is 5 × 10^7^. Therefore, the DER formation rate is 50%, which should also be observed. Unfortunately, it was not observed. All in all, the formation of DER is the result of stress induction dominated by orthogonal shear stress, but the reversal of shear stress direction is the necessary condition for its formation.

#### 3.4.2. WEA Formation

**Figure 10:**
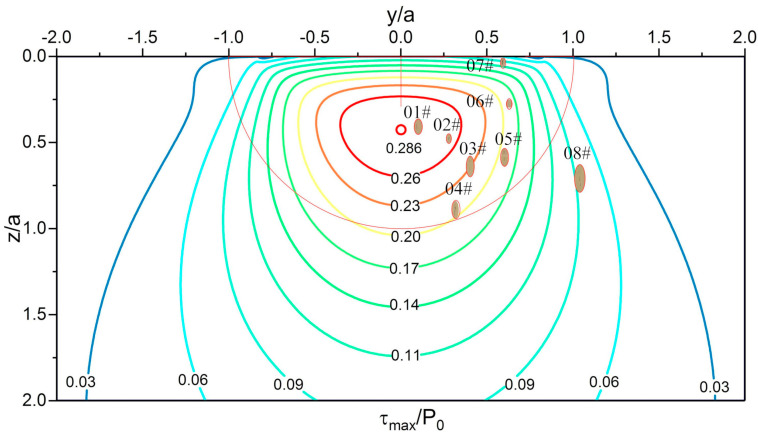
Composite image of inclusion positions and principal shear stress (τ_max_) field in 0.3 mm section of the plate specimen. Drawing of stress contours is based on Fu’s thesis [10]. For a point inside the material, y/a is the ratio of the horizontal distance (from the point to the contact center) to the contact radius, and z/a is the ratio of the depth to the contact radius.

WEA is a typical feature microstructure that appears during bearing service. Butterfly and WECs are both composed of nonmetallic inclusions and WEA, but they have two different morphologies. In this experiment, no butterfly or WECs were found, but the very early morphology of WEA was observed. Figure 10 is a composite figure of the positions of some inclusions in the material and the principal shear stress (τ_max_) field in 0.3 mm section. From this figure, the positions of inclusions and corresponding stress values can be intuitively seen.

**Figure 11:**
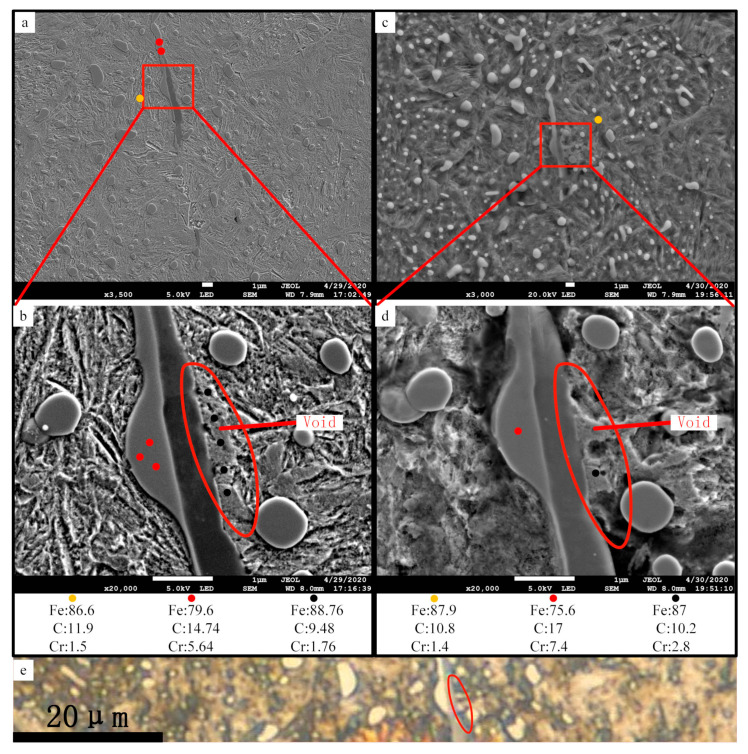
White etching area (WEA) adjacent to the inclusion observed in 0.3 mm section of the plate specimen. (**a**) Slightly etched micrograph of 05# inclusion; (**b**) detail view of (**a**) with EDS results; (**c**) deep etched micrograph of 05# inclusion; (**d**) detail view of (**c**) with EDS results; (**e**) optical micrograph of 05# inclusion after deep etching. The dots with different colours represent the detection position of EDS. Red is carbide, black is the WEA area, and yellow is the martensite matrix. Position of the inclusion is shown in Figure 10.

WEA was found in a 0.3 mm section at the interface of matrix and 05# inclusion. Figure 11a is the SEM micrograph of the specimen after slightly etched. Figure 11b is the partially enlarged view of (a). The surface morphology inside the red circle is obviously different from that of the matrix, and no martensite needle structure appears. Therefore, the specimen was exposed to secondary deep etching, and the corrosion resistance inside the red circle was observed. After the second etching, it was found that materials inside the red circle had obvious corrosion resistance compared with the matrix. Materials adjacent to inclusions are not all etched, as Figure 11b,d shows. No significant change was observed at the interface between the materials and the inclusion, before and after the secondary etching. However, other parts can clearly see the traces of material removal after secondary deep etching. In the field of optical microscope, a typical characteristic of WEA was found, as presented in Figure 11e. The target area shows obvious higher brightness.

After each etching, EDS detection was carried out at the WEA region and nearby area. It was found that the chemical composition of the WEA was similar to that of the matrix, which is not consistent with the characteristics of the WEA mentioned in the scientific reports. One of the reasons is that the minimum detection area of EDS equipment used in this experiment is 1 μm. When detecting the target area, carbon elements in the surrounding matrix or carbide are detected simultaneously. Although the absolute value of carbon EDS result is not accurate, the relative values of different positions still have comparative significance. According to the test results, the carbon content in the WEA region is basically the same as that in the matrix, just a little lower. In many scientific publications, carbon was not detected in the WEA. The reason is that carbon distributes in the boundary of WEA nanocrystals, and its scale exceeds the spatial resolution of EDS. However, it should be noted that the formation of WEA is a process of development, and Ooi [35] observed the initial morphology of WEA. It is characterised by the incomplete dissolution of carbides in the WEA region and the presence of microvoids on the observation surface. This is similar to the WEA observed in this experiment. The difference is that the size of WEA in Figure 11 is much smaller than the conventional size—less than micron level.

Grabulov [2,3,4] proposed the formation mechanism of butterfly according to the experimental phenomenon: under the cyclic stress, the inclusions separated from the matrix and interact with the matrix continuously, resulting in material transfer, forming a transfer layer with micro voids. Then the transfer layer crystallise into nano crystal, the voids converge into crack, and the WEA appear. In this process, cyclic stress induces dislocation movement, which leads to the migration of carbon elements and the formation of a nano cellular dislocation network [8,9,10,36], which makes it impossible for EDS to detect carbon elements. The debonding of inclusions and the matrix was also observed in [37]. In this experiment, it is also found that the inclusions were separated from the matrix, even in the original material. After cyclic compression load, the inclusions were more seriously separated from the matrix (see Section 3.4.3). Morsdorf [38] proved the existence of material transfer in the formation of WEA indirectly.

The target region observed in this paper, shown as Figure 11b,d, has the typical characteristic white colour under the light optical microscope, which can be determined as WEA. At the same time, it has the following characteristics: small scale, close to inclusions, voids in the interior and carbon can still be detected. It can be explained that it is the very initial form of WEA produced by the interaction between inclusions and matrix.

#### 3.4.3. Interaction of the Matrix and Inclusions

**Figure 12:**
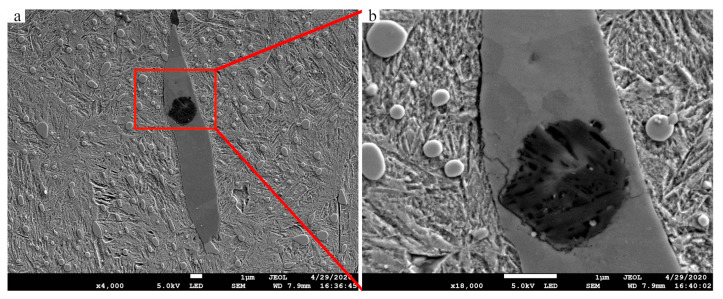
Internal crack of MnS inclusion in 0.3mm section of plate specimen. (**a**) 07# inclusion; (**b**) internal crack inside the 07# inclusion. Position of the inclusion is shown in Figure 10.

**Figure 13:**
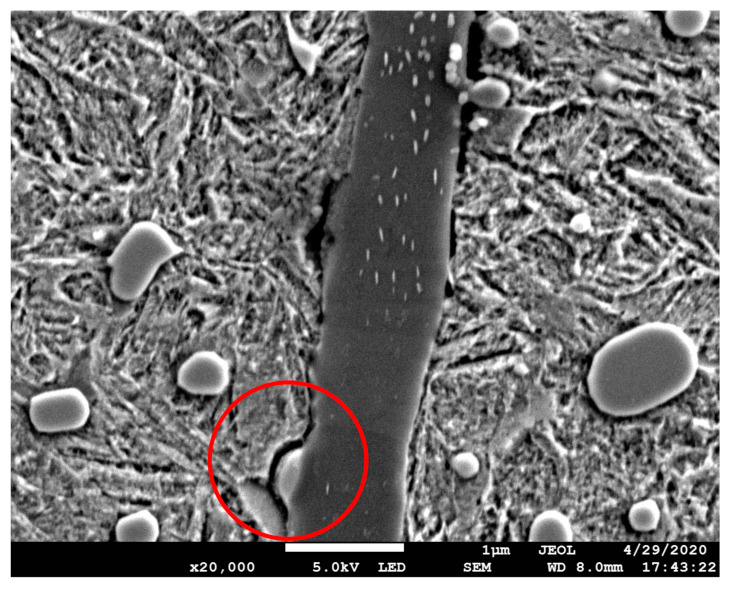
Crack formation near 08# inclusion in 0.3 mm section of plate specimen. Position of the inclusion is shown in Figure 10.

Internal crack was found in 07# MnS inclusion at 0.3 mm section of plate specimen and extended to the matrix interface (Figure 12). Crack is the precondition of WECs [1], it is the result of cumulative effect caused by cyclic compression load. In the material of wind turbine bearing after service, a butterfly-like microstructure starting from MnS was found [39]. Cracks frequently appear inside MnS inclusions, some of which produce WEA while others do not [37,39]. This similar phenomenon has been found in this paper, that is, there is no WEA-associated internal crack in MnS inclusions (Figure 12). Cracks also appeared at the interface between inclusions and the matrix, shown in Figure 13. However, the development of crack under cyclic compression conditions is much slighter than that under actual bearing service conditions. The main reason is that the actual service process of the wind turbine bearing is often accompanied by rolling slip, which will increase the internal material stress, and then accelerate the occurrence of cracks and WECs [5,15,18,40,41].

**Figure 14:**
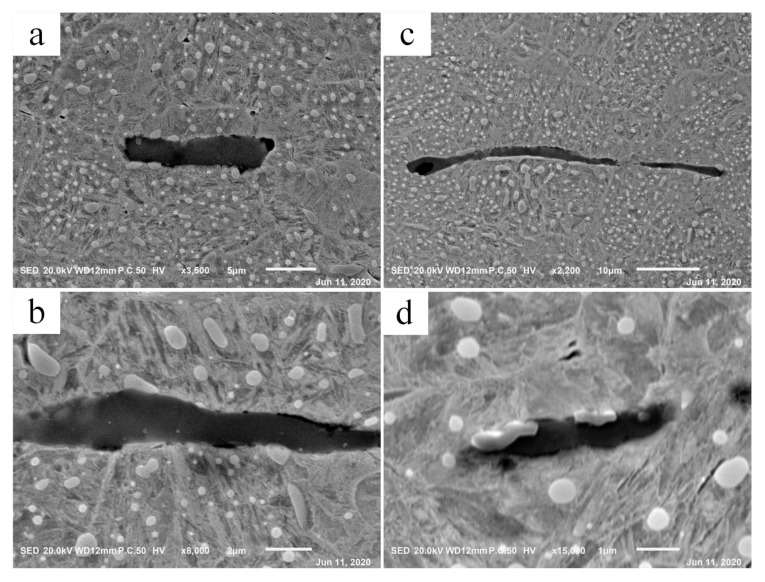
Local separation of inclusions from matrix in raw materials. (**a**–**d**) Specific examples observed by SEM.

**Figure 15:**
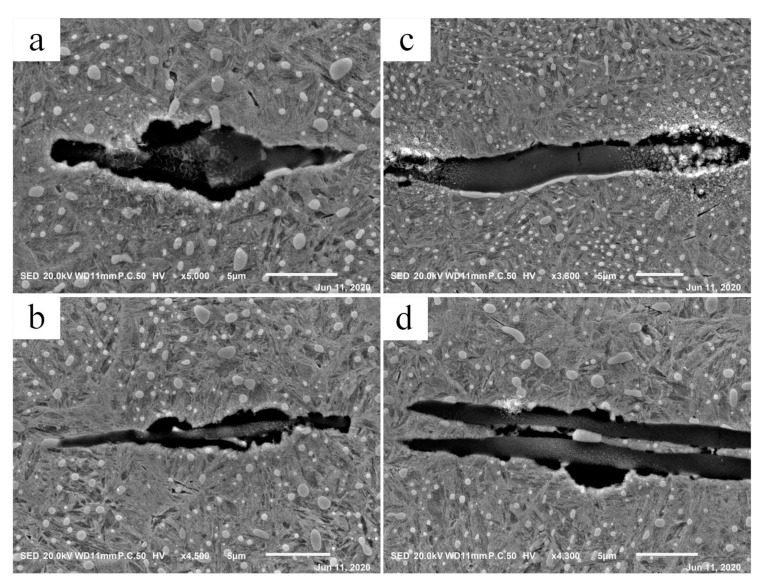
Separation of inclusions from matrix in cylindrical specimen. (**a**–**d**) Specific examples observed by SEM.

It is mentioned in the scientific reports that the separation of inclusions from the matrix is a necessary condition for the formation of WEA [4,37]. In this experiment, debonding was found in the original material; the interface between inclusions and the matrix presents a local separation, as shown in Figure 14. As a result of a higher coefficient of thermal expansion, MnS inclusions separated from the matrix during quenching, then new surfaces were generated, which were the initiation points of the cracks [42]. Figure 15 shows the interfaces after cyclic compression load in the cylinder specimen. The region of separation expanded and became more serious, obvious separation was observed at the interface.

**Figure 16:**
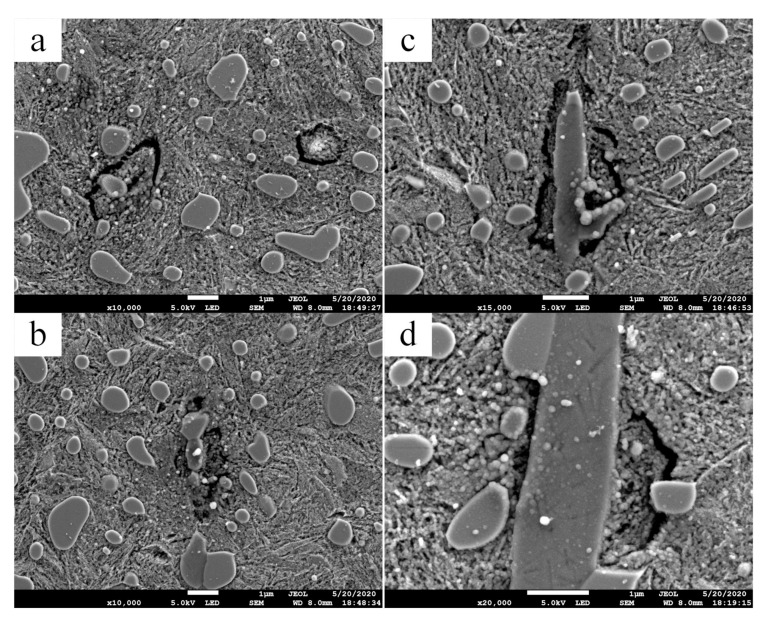
Transfer of materials observed inside the plate specimen in 0.0 mm section. (**a**) 03# inclusion; (**b**) 04# inclusion; (**c**) 05# inclusion; (**d**) 07# inclusion. Inclusions positions are shown in Figure 17.

**Figure 17:**
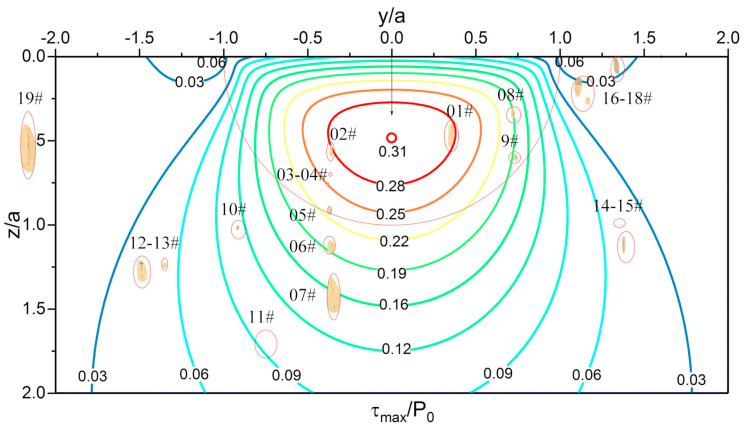
Composite image of inclusion positions and principal shear stress field in 0.0mm section of the plate specimen. Drawing of stress contours is based on Fu’s thesis [10]. For a point inside the material, y/a is the ratio of the horizontal distance (from the point to the contact center) to the contact radius, and z/a is the ratio of the depth to the contact radius.

In the plate specimen, as shown in Figure 16, the materials transferred to the inclusions and separated from the matrix, even near the carbide-matrix interface or completely inside the matrix. This phenomenon occurs near the maximum principal shear stress. Inclusions positions are shown in Figure 17. The surface of the separated part is not smooth in the etched state, and there is no difference between it and the matrix under SEM view, which indicates that it is not WEA.

As mentioned above, the formation mechanism of WEA is the transfer of matrix materials. Based on the separation between inclusions and matrix, the materials on both sides of the microgap contact with each other under cyclic stress and cause the transfer of matrix materials to inclusions. For the material separation without inclusions, the reason is that the actual material is not a strictly homogeneous material, there are defects or areas with poor binding in the material, and there will also be “hard points” with strong local binding, which play the role of inclusions. The phenomenon observed in this experiment is the material transfer under cyclic compression load. However, there is doubt that the scale of materials migration is the same as that of WEA presented in Figure 11, but no WEA is formed. In any case, the phenomenon that the material is separated from the matrix and transferred to the inclusions was found in the experiment, and the positions where this phenomenon occurs is closer to the maximum principal shear stress.

The tendency of inclusions separating from the matrix material was not observed in the plate-ball system, even in the 0.0 mm section where internal stress is the maximum. In the plate-ball system, the stress field produced by Hertz Contact is nonconforming, and the stress value is directly related with the position of inclusions. Only at some places inside the plate specimen, the maximum principal shear stress is the same as that of the cylindrical specimen, and at other places it is much smaller. In addition, orthogonal shear stress exists in the plate-ball system, but not in the cylinder specimen. These differences of internal stresses are the reasons why different results were observed in the two experimental systems.

## 4. Conclusions

The purpose of this paper is to eliminate the interference of many factors in the actual working condition of bearings and study the microstructural change caused by single factor (alternating stress field). In order to simulate the cyclic stress of bearing material in service, the cyclic compressive load was applied to the plate-ball system. A cylinder specimen, which bears a uniform stress field, was tested for comparison. The experiment has the following findings:

1. In the cylinder specimen, hardness increases slightly after undergoing cyclic compression load, and the degree of hardening is very low compared with the rolling contact condition. In the plate specimen, obvious hardening was observed. The dominant stress is orthogonal shear stress, and the degree of hardening is higher than that under rolling contact condition.
2. In the plate-ball system, oil film is produced locally in the contact area. The size of the oil film is smaller than that of the contact area, which indicates that some conditions need to be met for the production of oil film.
3. Although according to the calculation based on existing theory and mathematical model DER should arise, no DER was observed in the material. The reversal of stress is a necessary factor for the occurrence of DER.
4. In the plate-ball system, a very early morphology of WEA was found, which is produced by the interaction of the inclusion-matrix interface under cyclic compression stress.
5. In the cylinder specimen, the separation of inclusions from the matrix becomes worse. In the plate-ball system, materials transferred from the matrix to the inclusions.

## Figures and Tables

**Table 1:** Actual chemical composition of GCr15 used during experiment.

C	Mn	Si	Cr	Mo	Ni	Cu	S	P	Fe
0.967	0.37	0.214	1.45	0.0165	0.0475	0.0845	0.0045	0.0109	Balance
